# *Mycobacterium avium* Modulates the Protective Immune Response in Canine Peripheral Blood Mononuclear Cells

**DOI:** 10.3389/fcimb.2020.609712

**Published:** 2021-01-14

**Authors:** Suji Kim, Hyun-Eui Park, Woo Bin Park, Seo Yihl Kim, Hong-Tae Park, Han Sang Yoo

**Affiliations:** ^1^ Department of Infectious Diseases, College of Veterinary Medicine, Seoul National University, Seoul, South Korea; ^2^ BK21 FOUR Future Veterinary Medicine Leading Education and Research Center, Seoul National University, Seoul, South Korea; ^3^ Department of Microbiology, College of Medicine, Gyeongsang National University, Jinju, South Korea; ^4^ Department of Veterinary Physiology, College of Veterinary Medicine, Seoul National University, Seoul, South Korea; ^5^ Bio-MAX/N-Bio Institute, Seoul National University, Seoul, South Korea

**Keywords:** *Mycobacterium avium*, host immune response, Th17, apoptosis, dog

## Abstract

*Mycobacterium avium*, an opportunistic intracellular pathogen, is a member of the non-tuberculous mycobacteria species. *M. avium* causes respiratory disease in immunosuppressed individuals and a wide range of animals, including companion dogs and cats. In particular, the number of infected companion dogs has increased, although the underlying mechanism of *M. avium* pathogenesis in dogs has not been studied. Therefore, in the present study, the host immune response against *M. avium* in dogs was investigated by transcriptome analysis of canine peripheral blood mononuclear cells. *M. avium* was shown to induce different immune responses in canine peripheral blood mononuclear cells at different time points after infection. The expression of Th1-associated genes occurred early during *M. avium* infection, while that of Th17-associated genes increased after 12 h. In addition, the expression of apoptosis-related genes decreased and the abundance of intracellular *M. avium* increased in monocyte-derived macrophages after infection for 24 h. These results reveal the *M. avium* induces Th17 immune response and avoids apoptosis in infected canine cells. As the number of *M. avium* infection cases increases, the results of the present study will contribute to a better understanding of host immune responses to *M. avium* infection in companion dogs.

## Introduction

*Mycobacterium avium* is a member of the most common non-tuberculous mycobacteria complex that causes chronic respiratory disease in humans (Prevots and Marras, 2015; Yano et al., 2017). Although *M. avium* primarily infects humans and pigs, it has also been reported to infect several other mammalian species, such as cattle, sheep, horses, cats, and dogs (Pavlik et al., 2000; Campora et al., 2011). Other *Mycobacterium* species have been reported as common etiological agents of canine mycobacteriosis; however, dogs are known to be resistant to *M. avium* (Carpenter et al., 1988; Shackelford and Reed, 1989; Horn et al., 2000; Greene, 2006). Nonetheless, some type of breeds are more susceptible to *M. avium*, and an increasing number of cases of *M. avium* infection in dogs have been reported, several of which have shown granulomatous inflammation in infected organs, such as lung, liver, bone marrow, intestine and lymph nodes (Kim et al., 1994; Haist et al., 2008; Campora et al., 2011; Kim et al., 2016; Ghielmetti and Giger, 2020). The increase in such cases suggests the possibility of a potential public health risk attributable to *M. avium* infection in dogs. However, the mechanism underlying *M. avium* infection in dogs remains to be elucidated.

Typically, the host immune response attempts to defend against *M. avium* together with macrophages and T lymphocytes early during an infection. T cell immune responses are important in regulating pulmonary *M. avium* complex (MAC) infection, with T helper 1 (Th1) responses playing an important role in increasing macrophage bactericidal capacity, while T helper 17 (Th17) differentiation induces neutrophilic pulmonary inflammation (Matsuyama et al., 2014; Shu et al., 2018). Th1 cells eradicate mycobacteria by producing various cytokines (Haverkamp et al., 2006). Tumor necrosis factor (TNF) induces antigen-specific CD4^+^ cells that produce IFN-γ early during an infection. IFN-*γ* is well known to limit *Mycobacterium* infection by inhibiting outgrowth (Patel et al., 2005). These cytokines are essential for protecting MAC early during an infection by developing cell-mediated immune responses.

Th17 cells are important for establishing a protective immune response to *Mycobacterium* (Kozakiewicz et al., 2013). Th17 cells can accumulate Th1 cells in infected tissues and enhance the antimycobacterial response with Th1 cells (Weaver et al., 2013; Cruz et al., 2015b). Th17 cells produce the lineage-specific cytokines IL-17A and IL-17F as well as other cytokines (IL-6 and GM-CSF) and chemokines (CXCL1, CXCL2, CXCL5, and CXCL8) (Jasenosky et al., 2015; Lombard et al., 2016). Importantly, IL-17 enhances the migration of neutrophils to the inflamed sites for the early clearance of bacteria by inducing CXC chemokines during *Mycobacterium* infection. However, Th17 cells have a pathological role rather than a protective role under Th1-diminished conditions after *M. avium* infection (Matsuyama et al., 2014; Xu et al., 2016). In particular, IL-17 plays crucial roles in chronic inflammation and is important for the formation and maintenance of granulomas in mycobacterial infection sites (Ostadkarampour et al., 2014; Li et al., 2016). IL-1β, IL-6, and IL-23 induce the Th17 pathway and form granulomas with IL-17 (Stark et al., 2005). In addition, IL-17 contributes to the persistence of *M. avium* in macrophages *via* the NF-*κ*B and MAPK signaling pathways (Vázquez et al., 2012). These cytokines are important for the immune response to chronic pulmonary *Mycobacterium* infection.

*M. avium* is an intracellular pathogen that primarily affects macrophages (Thegerström et al., 2012), where infected macrophages undergo apoptotic cell death to minimize tissue injury and decrease pathogen viability (Behar et al., 2010). However, *M. avium* survives intracellularly and replicates within macrophages by preventing the phagosome maturation process (Early et al., 2011). *Mycobacterium* inhibits the apoptosis of macrophages *via* several mechanisms involving TNF-, caspase-, NO-, and cathepsin-related mechanisms (Rojas et al., 1998; Chen et al., 2006; O’Sullivan et al., 2007). In particular, *M. avium* has been reported to inhibit bacterial programmed cell death induced by both the extrinsic pathway through caspase 8 activation and the intrinsic apoptotic pathway through caspase 3 activation (Sharbati et al., 2011; Kabara and Coussens, 2012). Furthermore, IL-17A has also been reported to be associated with the inhibition of apoptosis by a p53-dependent mechanism during *Mycobacterium* infection (Cruz et al., 2015a).

As is the case for *M. avium* infection of several mammalian species, the host response of infected dogs should be studied to estimate the possibility of *M. avium* infection. In the present study, we elucidated the host responses in canine peripheral blood mononuclear cells and monocyte-derived macrophages upon infection with *M. avium*. Our results revealed that the T cell response shifts from a Th1 to a Th17 cell response according to the time of infection and that the expression of apoptosis-related genes decreased as intracellular *M. avium* proliferates in macrophages. The results of the present study will promote a better understanding of the host immune responses to *M. avium* in dogs and highlight the potential risk of mycobacterial infections in various species.

## Materials and Methods

### Bacterial Strains and Cultivation

*M. avium* subsp. *hominissuis* strain 104 was kindly provided by Prof. SJ Shin from the College of Medicine, Yonsei University in Seoul, Korea. *M. avium* was cultured on Middlebrook 7H11 agar supplemented with OADC (BD Biosciences, CA, USA). After 7 days, the cells were cultured in Middlebrook 7H9 broth for 5 days. Cultures at an optical density of 0.45 at 600 nm (9.2 × 10^8^ CFU/ml) were generated after vigorous vortexing for 30 s to remove clumps.

### Blood Cell Isolation

Blood samples were collected from six healthy Beagle dogs in accordance with the Guide for the Care and Use of Laboratory Animals and the Animal Welfare Act in the animal facility of the 2^nd^ Research Center at Genia (Eumsung, Korea). Blood was collected from unanesthetized dogs by professional veterinarians with permission approved by the Institutional Animal Care and Use Committee (IACUC) at Genia (IACUC number; ORIENT-IACUC-19026). Whole blood was diluted 1:3 in RPMI 1640 (Gibco, NY, USA) containing 20% of inactivated fetal bovine serum (FBS; Gibco) and added to a gradient with 1.077 g/ml of histopaque (Sigma Aldrich, Taufkirchen, Germany). Peripheral blood mononuclear cells (PBMCs) were collected *via* density gradient centrifugation (400 × g for 30 min) using leucoseptube (Greiner Bio-One, Kremsmünster, Austria). Then, the PBMCs were washed twice with DPBS containing 5% FBS, 1% penicillin/streptomycin, and heparin (2,000 U/ml) and centrifuged at 250 × g for 5 min, after which the cells were resuspended in RPMI 1640 containing 20% FBS and 1% penicillin/streptomycin and cultured for 24 h at 37°C.

### Cell Culture and Polarization

PBMCs were seeded into 24-well plates (ThermoScientific, MA, USA) and cultured for 12 h in RPMI 1640 supplemented with 10% FBS, after which they were used for RNA-Seq analysis after *M. avium* stimulation. The protocol described by Goto-Koshino, Yuko, et al. was used to culture canine macrophages from blood-derived monocytes (Goto-Koshino et al., 2011). Adherent cells that strongly adhered to the plastic base of flasks were considered monocytes and collected (Delirezh et al., 2013; Heinrich et al., 2017). Canine monocytes were stimulated with 1 µg of PMA to induce macrophage differentiation for an additional 6 days. Then, canine monocyte-derived macrophages (MDMs) were seeded into the wells of plates (ThermoScientific) containing the same medium supplemented with 10% FBS to stabilize the cells.

### RNA Sequencing

Canine PBMCs were infected with *M. avium* at a multiplicity of infection (MOI) of one with DPBS added to one plate as a negative control. Total RNA was isolated at 0, 6, 12, and 24 h after stimulation using an RNeasy Mini kit (Qiagen, Hilden, Germany). After the quality of isolated RNA was assessed using RNA 6000 Nano Chip with an Agilent 2100 bioanalyzer (Agilent Technologies, Amstelveen, The Netherlands), RNA libraries were constructed using a QuantSeq 3′mRNA-Seq Library Prep kit (Lexogen, Inc., Austria). Total RNA was hybridized with an oligo-dT primer including an Illumina-compatible sequence at its 5′ end and cDNA library was synthesized using a random primer. The double-stranded library was amplified with the complete adapter sequences and the PCR product was purified. High-throughput sequencing was performed *via* single-end 75 sequencing using a NextSeq 500 instrument (Illumina Inc., CA, USA).

QuantSeq 3′mRNA-Seq reads were aligned using the index of Bowtie2 (Langmead and Salzberg, 2012), which is generated by aligning genome assembly sequences or representative transcript sequences to genome or transcriptome, and the alignment was also used for the estimation of transcriptional abundance. Differentially expressed genes were determined by counting the reads on the unique and multiple alignments using BEDTools (Quinlan and Hall, 2010) and the read count was processed by quantile normalization method using EdgeR within R (Team, R Core, 2016). Functional genes were classified by DAVID (http://david.abcc.ncifcrf.gov/) and Medline databases (http://www.ncbi.nlm.nih.gov/). Pathway analysis was performed by Ingenuity Pathway Analysis (Qiagen Inc., https://www.qiagenbioinformatics.com/products/ingenuitypathway-analysis) (Krämer et al., 2013).

### Quantification of Gene Expression

RNA-Seq data was validated by RT-qPCR and the correlationa coefficient between the two analyses was 0.9024 (**Supplementary Figure 1**). cDNA was synthesized using a QuantiNova Reverse Transcription Kit (Qiagen), and RT-qPCR was performed using a Rotor-Gene SYBR Green PCR kit (Qiagen). The genes were amplified with a Rotor-Gene Q real-time PCR cycler (Qiagen). Amplification conditions were described in **Supplementary Table 1**. The gene expression levels were determined *via* the 2^−ΔΔCt^ method with glyceraldehyde-3-phosphate dehydrogenase (GAPDH) as a reference gene. The fold change was determined based on the relative gene expression level compared to the control.

### Caspase Activity Assay

Canine monocyte-derived macrophages were stimulated with *M. avium* at an MOI of 1:1 for 6, 12, and 24 h. Caspase activity was monitored by measuring the active forms of caspase 3 and caspase 7 with the Caspase-Glo^®^ 3/7 Assay System (Promega, WI, USA) according to the manufacturer’s protocol. To identify the activity of caspases after the induction of apoptosis, cells were treated with hydrogen peroxide (H_2_O_2_) for 30 min before 24 h of infection with *M. avium*, which is known to stimulate caspase activity (Jones et al., 2000; Kabara and Coussens, 2012). Treatment with 100 µm H_2_O_2_ for 30 min was used based on time course and dose–response curve studies with uninfected MDMs. Each group was assayed with additional control samples, including cell medium, reagent, *M. avium* and negative control to calculate the RLU values.

### Invasion and Proliferation Assay

Bacterial invasion assays with canine monocyte-derived macrophages were performed as described by Bermudez and Sangari (Bermudez and Young, 1994; Sangari et al., 2000). Canine monocyte-derived macrophages were infected for 2 h with *M. avium* at an MOI of one. After centrifugation at 400 × g for 5 min, the cells were washed with DPBS and treated with amikacin at a concentration of 200 µg/ml for 2 h to kill extracellular bacteria (Bermudez and Young, 1994; Sangari et al., 2000). The cells were incubated for 4, 12, and 24 h, after which they were washed, and the viable intracellular bacteria were released by incubation after treatment of 1% Triton X-100 (Sigma-Aldrich, MO, USA). Then, the samples were vigorously vortexed and agitated for 30 s to lyse cells. Bacteria were serially diluted and then plated onto 7H11 agar plates to enumerate viable bacteria.

### Quantification of Cytokines

Canine IL-17, IL-6, IL-10, IL-12, IL-4, IL-1β, and IFN-*γ* were detected in the supernatants of canine peripheral blood mononuclear cells at 24 h post *M. avium* infection using DuoSet^®^ and Quantikine^®^ ELISA kits (R&D Systems, Minneapolis, MN, USA), according to the manufacturer’s instruction.

### Statistical Analysis

Statistical significance was analyzed by Student’s t-test using GraphPad Prism version 7.00 (Windows, GraphPad Software, La Jolla California USA, www.graphpad.com). Significantly expressed genes were determined at *p <*0.05. Fold changes are represented by the mean ratio of gene expression in *M. avium*-infected cells/uninfected cells.

## Results

### Characterization of Canine Immune Responses Against *Mycobacterium avium* Infection by Differentially Expressed Genes

**Figure 1:**
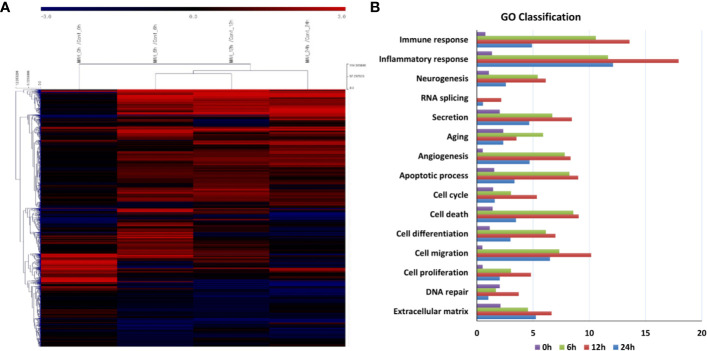
Gene expression analysis of canine peripheral blood mononuclear cells infected with *Mycobacterium avium* at 0, 6, 12, and 24 h post infection. **(A)** Clustering analysis and **(B)** GO analysis of DEGs in *M. avium* infected-canine PBMCs (|*Fold change*| ≥ 2.0, normalized data (log2) = 4, *p*-value <0.05).

The transcriptomes of canine PBMCs infected with *M. avium* for 0, 6, 12, and 24 h were analyzed by RNA-Seq. Sixteen cDNA libraries from uninfected and *M. avium*-infected cells were sequenced. Approximately 92.87% of clean reads were uniquely mapped onto the canFam 3. A total of 3,366 differentially expressed genes (DEGs) were significantly expressed in canine PBMCs-infected with *M. avium* compared to the uninfected group (|*fold change*| ≥ 2.0, normalized data (log2) = 4, *p*-value <0.05). The DEGs from the cells infected with *M. avium* for 6 and 12 h clustered, while those from cells infected for 24 h were separated from the other groups (**Figure 1A**). Most DEGs belonged to the GO term categories immune response and inflammatory response compared with 0 h-infection. The percentage of significant DEGs-annotated immune response in the groups infected for 6, 12, and 24 h were 10.57, 13.58 and 4.91%, respectively. The percentage of inflammatory response was 11.66, 17.94, and 12.11% at 6, 12, and 24 h.p.i (**Figure 1B**).

The comparison analysis of canonical pathways showed that signaling pathways were expressed in relation to cellular immune responses against *M. avium* infection (**Table 1**). The pathways related to the Th1 response (HMGB1 Signaling, Neuroinflammation Signaling pathway, TREM1 Signaling, MIF-mediated Glucocorticoid Regulation, Dendritic Cell Maturation, and Type I Diabetes Mellitus Signaling) were activated at 6, 12 and 24 h after *M. avium* infection. The Th17 response-related pathways (IL-6 Signaling, IL-23 Signaling Pathway, Role of IL-17F in Allergic Inflammatory Airway Diseases, Th17 Activation Pathway, LXR/RXR Activation, and PPAR Signaling) were commonly expressed at all infection times. Molecules associated with the inhibition of apoptosis were expressed in the pathways Small Cell Lung Cancer Signaling, B Cell Receptor Signaling, and Interferon Signaling.

**Table 1:** Comparison analysis of canonical pathways in *Mycobacterium avium*-infected canine peripheral blood mononuclear cells at 0, 6, 12, and 24 h.

Canonical Pathways	0h	6h	12h	24h
PPAR Signaling	1.633	−4.747	−3.651	−2.058
Dendritic Cell Maturation	−1.569	4.529	3.452	1.414
TREM1 Signaling	−1.265	3.71	3.578	2.353
LXR/RXR Activation	1	−1.897	−3.053	−3.742
Th17 Activation Pathway	−0.333	3.71	3.441	2.117
IL-6 Signaling	−0.378	3.781	3.124	2.271
Role of IL-17F in Allergic Inflammatory Airway Diseases	−0.447	3	3.051	2.673
Neuroinflammation Signaling Pathway	0.707	4.906	2.252	1.27
HMGB1 Signaling	1	2.92	3.413	1.718
Phospholipase C Signaling	1.961	2.197	2.694	−1.414
Type I Diabetes Mellitus Signaling	0.447	2.746	2.5	2.5
Interferon Signaling	–	3	2.714	2
Osteoarthritis Pathway	−0.756	4.025	2.449	−0.471
Cardiac Hypertrophy Signaling (Enhanced)	−0.367	3.763	2.251	−1.315
Small Cell Lung Cancer Signaling	0.816	2.121	2.121	2.53
Colorectal Cancer Metastasis Signaling	−0.174	2.219	2.546	−2.458
IL-23 Signaling Pathway	−0.378	1.941	2.138	2.828
B Cell Receptor Signaling	1.89	2.121	2.694	0.507
Synaptogenesis Signaling Pathway	1.622	1.021	0.289	−4.249
MIF-mediated Glucocorticoid Regulation	1.134	2.309	2.714	1

Canonical pathways are indicated with the z-score from the pathway activation analysis.

Proinflammatory cytokines and molecules related to Th1 cells (TNF-α, IL-8, IFN-*γ*, IL-1β, TREM1, and PTGS2) were upregulated in the pathways HMGB1 Signaling, Neuroinflammation Signaling Pathway, TREM1 Signaling, Type I Diabetes Mellitus Signaling, MIF-mediated Glucocorticoid Regulation, and Dendritic Cell Maturation after 6, 12, and 24 h post infection. Molecules related to the Th17 immune responses (IL-6, IL-23, IL-17A, IL-17F, ROR*γ*T, and IL22) were also commonly upregulated in the following signaling pathways; IL-6 Signaling, IL-23 Signaling Pathway, Role of IL-17F in Allergic Inflammatory Airway Diseases, and Th17 Activation Pathway. PPARG, RXRA, NR1H3, and NR1H4, as nuclear receptors that affect the inhibition of Th17 differentiation, were downregulated in the pathways LXR/RXR Activation and PPAR Signaling. In the Small Cell Lung Cancer Signaling pathway, the molecules BIRC2, BCL2L1, and TRAF, which were commonly upregulated at 6, 12, and 24 h, were associated with the inhibition of apoptosis. In relation to apoptosis inhibition, IFI6 of Interferon Signaling and molecules-related to the PI3K/AKT pathways of B Cell Receptor Signaling were commonly upregulated at 6, 12, and 24 h.

### Activation of Signaling Pathways Related to the Cellular Immune Response Against *Mycobacterium avium* Infection

The top 20 canonical pathways showed that canine immune responses changed over time in response to *M. avium* infection. Significant signaling pathways [–*log*(*p* – *value*) ≥ 1.3] were related to both the Th1 and Th17 responses at 6 and 12 h, while pathways at 24 h were related to the Th17 immune response. Then, after 6 h of *M. avium* infection, Th1 cell-related pathways (HMGB1 Signaling, Acute Phase Response Signaling, and NF-*κ*B Signaling) were activated. In addition, Th17 immune response-related pathways (LXR/RXR Activation, Role of Macrophages, Fibroblasts ad Endothelial Cells in Rheumatoid Arthritis, IL-6 Signaling, STAT3 Signaling, LPS/IL-1 Mediated Inhibition of RXR Function, and PPAR Signaling) were also activated (**Supplementary Table 2**).

In canine PBMCs infected with *M. avium* for 12 h, signaling pathways related to Th1 immune responses were activated (HMGB1 Signaling, Acute Phase Response Signaling, Role of Pattern Recognition Receptors in Recognition of Bacteria and Viruses, Toll-like Receptor Signaling, and Hepatic Fibrosis/Hepatic Stellate Cell Activation). In addition, Th17 cell response-related signaling pathways (The pathways Role of Macrophages, Fibroblasts and Endothelial Cells in Rheumatoid Arthritis, IL-6 Signaling, Differential Regulation of Cytokine Production in Macrophages and T Helper Cells by IL-17A and IL-17F, Role of Hypercytokinemia/hyperchemokinemia in the Pathogenesis of Influenza, Role of Osteoblasts, Osteoclasts and Chondrocytes in Rheumatoid Arthritis, Altered T Cell and B Cell Signaling in Rheumatoid Arthritis, LXR/RXR Activation, and Differential Regulation of Cytokine Production in Intestinal Epithelial Cells by IL-17A and IL-17F) were also activated (**Supplementary Table 3**).

Signaling pathways expressed at 24 h post infection were associated with the Th17 immune response. In addition, the pathways related to Th17 immune response (Role of Osteoblasts, Osteoclasts and Chondrocytes in Rheumatoid Arthritis, Role of Macrophages, Fibroblasts and Endothelial Cells in Rheumatoid Arthritis, Differential Regulation of Cytokine Production in Macrophages and T Helper Cells by IL-17A and IL-17F, Colorectal Cancer Metastasis Signaling, Differential Regulation of Cytokine Production in Intestinal Epithelial Cells by IL-17A and IL-17F, Role of Cytokines in Mediating Communication between Immune Cells, and Role of Hypercytokinemia/hyperchemokinemia in the Pathogenesis of Influenza) were activated. Furthermore, signaling pathways related to apoptosis (LXR/RXR Activation, LPS/IL-1 Mediated Inhibition of RXR Function, and FXR/RXR Activation) were inhibited in the canine PBMCs infected with *M. avium* for 24 h (**Supplementary Table 4**).

### Increase of Th17-Related Molecules in Canine Peripheral Blood Mononuclear Cells Infected With *Mycobacterium avium*

**Figure 2:**
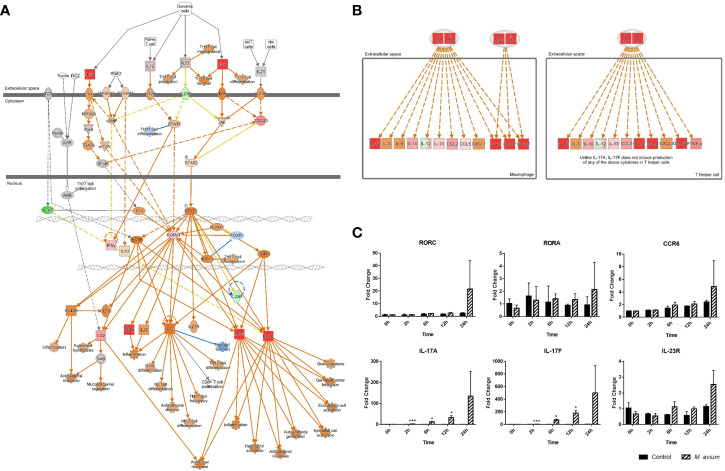
Activation of Th17 pathways in canine peripheral blood mononuclear cells infected with *Mycobacterium avium*. **(A)** Ingenuity pathway analysis of ‘Th17 Activation Pathway’ at 12 h.p.i. and **(B)** ‘Differential Regulation of Cytokine Production in Macrophages and T Helper Cells by IL17A and IL17F’ at 24 h.p.i. Individual nodes represent proteins with relationships represented by edges. The genes shown in red indicate upregulation, green indicates downregulation, orange indicates predicted activation, and an uncolored node indicates that the genes were not differentially expressed in this pathway. **(C)** Gene expression levels of Th17 pathways during *M. avium* infection. RORC, RORA, IL-17A, IL-17F, IL-23R, and CCR6 were indicated by an mRNA fold-change in canine PBMCs infected with *M. avium*. mRNA expression in uninfected cells at 0 h was considered 1 as a reference for fold-change in expression. *p < 0.05 and ***p < 0.001.

Among the Th17-related signaling pathways, ‘Th17 Activation Pathway’ and ‘Differential Regulation of Cytokine Production in Macrophages and T Helper Cells by IL17A and IL17F’ were commonly activated in canine PBMCs at all times of infection (**Figures 2A, B**). In particular, the molecules associated with Th17 immune responses (CSF2, IL22, IL17A, and IL17F) were highly expressed in the Th17 Activation pathway after 24 h.p.i. (**Supplementary Table 5**). Regarding Differential Regulation of Cytokine Production in Macrophages and T Helper Cells by IL17A and IL17F, the molecules (CCL3, CCL4, CSF2, CSF3, IL17A, and IL17F) were activated after 24 h.p.i. (**Supplementary Table 6**). The key genes of Th17 pathways including transcription factors (RORC and RORA), chemokine (CCR6), cytokines (IL-17A, IL-17F, and IL-23R) were increased after time of *M. avium* infection (**Figure 2C**).

**Figure 3:**
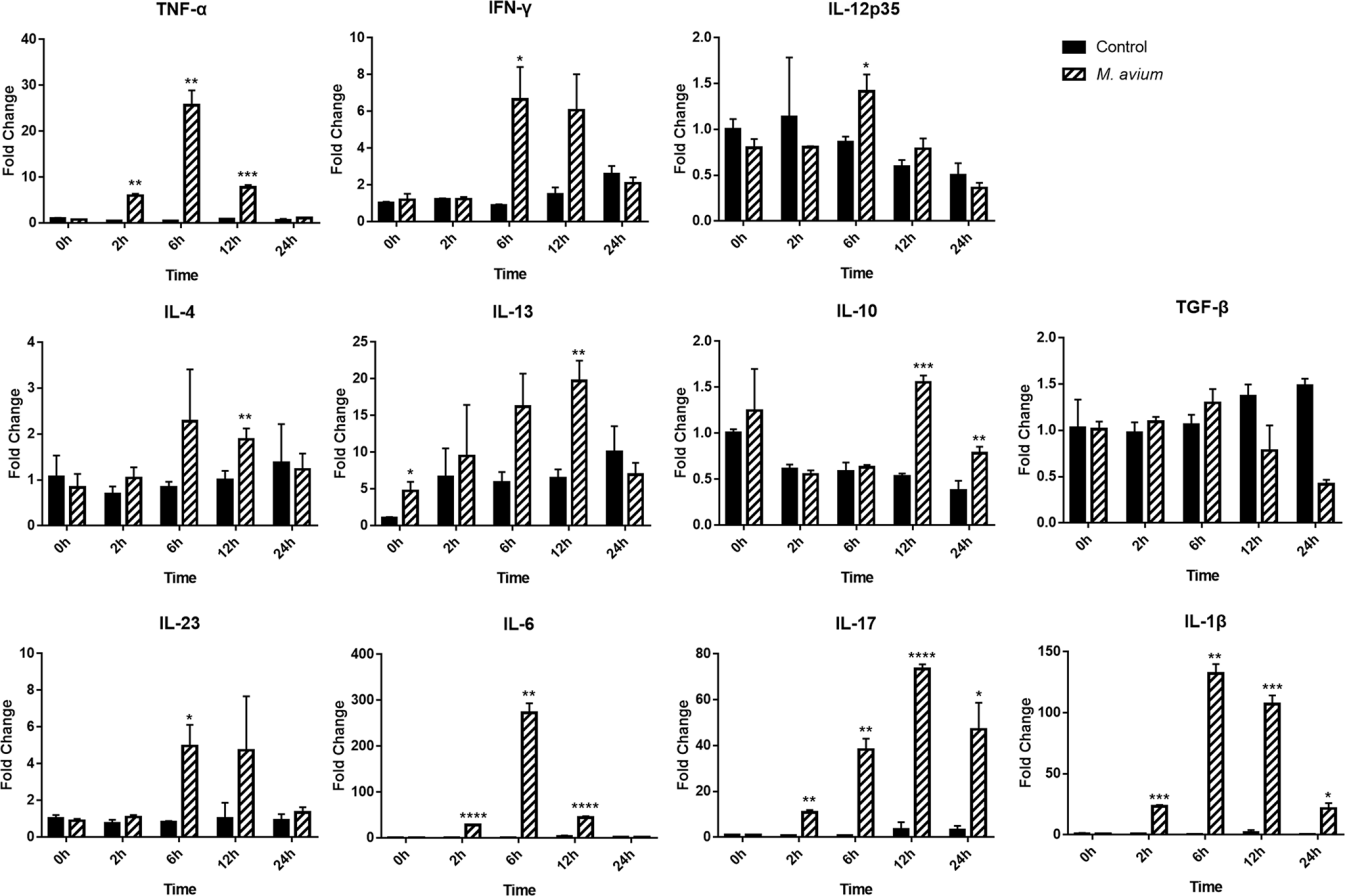
Different cytokine mRNA expression in *Mycobacterium avium*-infected canine peripheral blood mononuclear cells. Quantification of the cytokines IL12p35, IFN-*γ*, and TNF-α (Th1-type), IL-4, and IL-13 (Th2-type), IL-1*β*, IL-6, IL-17, and IL-23 (Th17-type), and IL-10 and TGF-*ß* (T regulatory-type), as indicated by an mRNA fold-change in canine PBMCs infected with *M. avium*. Cytokine mRNA expression in uninfected cells at 0 h was considered 1 as a reference for fold-change in expression. *p < 0.05, **p < 0.01, ***p < 0.001, and ****p < 0.0001.

The patterns of cytokines observed by gene expression analysis in canine PBMCs showed they were related to the Th1 and Th17 immune responses. The expression of genes related to Th1-related cytokines (TNF-α, IFN-*γ*, and IL-12p35) and Th17-related cytokines (IL-23 and IL-6) were significantly increased in canine PBMCs after 6 h post infection. The expression of genes related to Th2-related cytokines (IL-4 and IL-13) and Treg-related cytokines (IL-10) were slightly increased at 12 h, while Th17-related cytokines (IL-1β and IL-17) were highly increased (Fold change; 107.05 ± 7.12 and 73.37 ± 2.04) at that time (**Figure 3**). The quantification of cytokines (IL-17, IL-1β, IL-6, IL-10, IL-4, IL-12, and IFN-γ) was measured by ELISA from supernatant of canine PBMCs infected with *M. avium* (**Supplementary Figure 2**). The results also showed that IL-17 and IL-1β were highly expressed (concentration; 4642.87 ± 604.14 pg/ml and 1566.33 ± 252.73 pg/ml) at 24 h.p.i.

### Inactivation of Apoptosis Signaling and Intracellular Replication of *Mycobacterium avium* in Infected Cells

**Figure 4:**
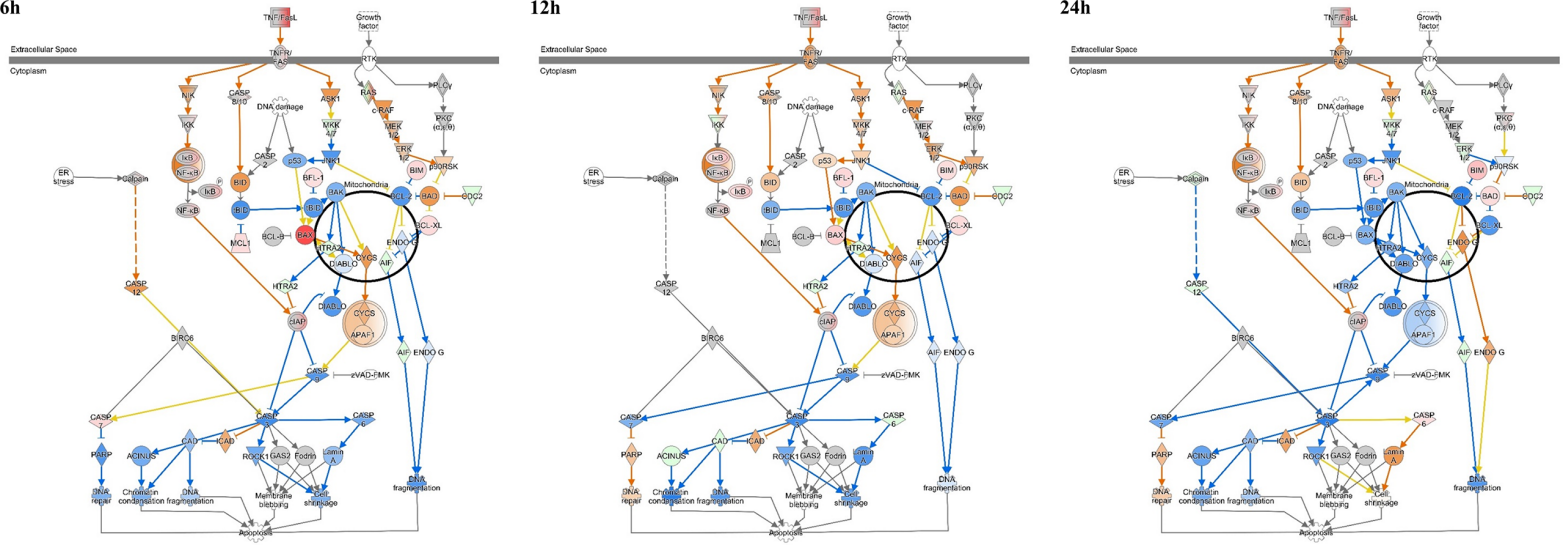
Ingenuity pathway analysis of ‘Apoptosis Signaling’ in *Mycobacterium avium*-infected canine peripheral blood mononuclear cells for 6, 12, and 24 h. Individual nodes represent proteins with relationships represented by edges. The genes shown in red indicate upregulation, green indicates downregulation, orange indicates predicted activation, blue indicates predicted inhibition, and an uncolored node indicates that the genes were not differentially expressed in this pathway.

**Figure 5:**
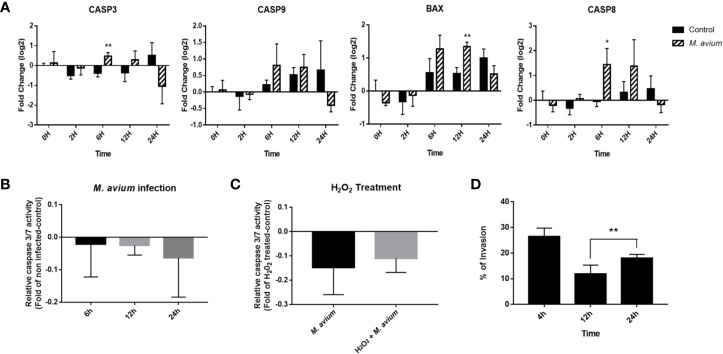
Analysis of apoptosis signaling in *Mycobacterium avium* infection. **(A)** Expression profiling of apoptosis-related genes in *M. avium*-infected canine PBMCs. Quantification of mRNA related to apoptosis, as represented by an mRNA fold-change in canine PBMCs infected with *M. avium*. The mRNA expression in noninfected cells at 0 h was considered 1 as a reference for the fold-change in expression. **(B)** Caspase 3/7 activity in canine monocyte-derived macrophages infected with *M. avium*. The fold-change was calculated between the cells infected with and without *M. avium*. **(C)** Caspase 3/7 activity in *M. avium*-infected MDMs with or without apoptosis induction. The fold-change was calculated between the *M. avium*-infected cells treated with or without 100m H_2_O_2_ and the apoptosis-induced cells. **(D)** The invasion and replication ability of *M. avium* in canine monocyte-derived macrophages. Graph showing the percentage of intracellular *M. avium* cells in MDMs and after treatment with amikacin. Each column represents the mean ± SD of nine independent experiments. *p < 0.05, **p < 0.01.

Apoptosis signaling was inhibited in canine PBMCs after 6, 12, and 24 h post infection (z-score = −.0.408, −1.043, and −1.633). In particular, the BAX-CYCS-CASP9-CASP3/CASP7 pathways were inactivated after 24 h.p.i. (**Figure 4**). Regarding the mRNA abundance for genes in this pathway, *caspase 3*, *caspase 8*, *caspase 9*, and *bax* were increased until 12 h.p.i.; however, they were downregulated after that time (**Figure 5A**). The activities of caspase 3/7 in canine monocyte-derived macrophages (MDMs) infected with *M. avium* decreased slightly over the course of infection compared to the uninfected cells (**Figure 5B**). To determine whether *M. avium* was affected by the apoptosis of macrophages, we measured the activities of caspase 3/7 after induction of apoptosis with H_2_O_2_. Both MDMs infected with *M. avium* after H_2_O_2_ treatment and *M. avium*-infected MDMs without H_2_O_2_ showed they lowered the activities of caspase3/7 compared to the uninfected cells treated with H_2_O_2_ (**Figure 5C**). Cell invasion was measured by enumerating intracellular bacteria after amikacin treatment to kill extracellular bacteria. *M. avium* replicated in canine MDMs after invasion (**Figure 5D**), where the percentage of intracellular *M. avium* was 26.5 ± 3% in canine MDMs after 4 h.p.i. After invasion, the number of intracellular *M. avium* significantly increased in canine MDMs (*p* < 0.001) after 24 h post infection. The number of intracellulare *M. avium* was shown in **Supplementary Figure 3** (4 h; 26,450 ± 3,256 CFU/ml, 12 h; 11,800 ± 3,527 CFU/ml, and 24 h; 17,955 ± 1,542 CFU/ml).

## Discussion

As the global incidence of non-tuberculous mycobacterial infection increases, *Mycobacterium avium* complex (MAC) organisms have been increasingly isolated from various hosts (Inderlied et al., 1993; Martín-Casabona et al., 2004). In particular, *M. avium*, which causes chronic pulmonary disease, has been isolated from several mammals (Huchzermeyer and Michel, 2001). *M. avium* infection has been reported in a wide range of animals, including companion dogs and cats (Pavlik et al., 2000; Campora et al., 2011). Disseminated *M. avium* infection in dogs has been consistently reported, and most cases report granulomatous inflammation in infected organs (Horn et al., 2000; Campora et al., 2011; Lam et al., 2012; Kim et al., 2016; Ghielmetti and Giger, 2020). As the number of cases of *M. avium* infection in dog increases, understanding the mechanisms of *M. avium* infection is necessary to prevent potential mycobacterial infection. In the present study, we analyzed host immune responses against *M. avium* infection in canine peripheral blood mononuclear cells by transcriptome analysis.

Transcriptomic analysis of canine immune responses to *M. avium* showed that they were related to the activation of the Th1 and Th17 immune responses and the inhibition of apoptosis. The hierarchical clustering analysis showed that these immune responses were clustered depending on the time of infection. At an early time of infection, both Th1 and Th17 immune responses were activated, while signaling pathways expressed at 24 h were associated with the Th17 immune response. An analysis of signaling pathways also showed that they were related to the inhibition of apoptosis.

T cell immunity regulates pulmonary *M. avium* infection, with Th1 and Th17 responses being particularly essential during *M. avium* infection (Matsuyama et al., 2014). Th1 immune responses play a critical role in mycobactericidal activities early during an infection. Th1 responses are important for the clearance of mycobacteria through the production of cytokines (Patel et al., 2005; Thegerström et al., 2012). IFN-*γ* inhibits mycobacterial growth by IFN regulatory factors induced by infection (Vila-del Sol et al., 2008). TNF-α plays a key role in increasing host resistance to *Mycobacterium* infection during the Th1 response (Keane et al., 2001). In the present study, commonly expressed signaling pathways showed that Th1 immune response-related molecules (TNF-α, IL-8, IFN-*γ*, IL-1β, TREM1, and PTGS2) were activated. Furthermore, the observed abundances of mRNA related to T cell responses also indicated Th1 cell-related molecules (TNF-α and IFN-*γ*) were significantly activated early in an infection.

Th17 cells play a role in antimycobacterial immunity to mycobacterial infections, accelerating the accumulation of Th1 cells (Gopal et al., 2012). IL-23, IL-6, and IL-1β produced by antigen presenting cells induce the Th17 pathway (Shu et al., 2018). Th17 lineage cytokines (IL-17A, IL-17F, and IL-22) and chemokines (CXCL1, CXCL2, CXCL5, and CXCL8) are known to control chronic lung infection caused by mycobacteria (Busman-Sahay et al., 2015; Lombard et al., 2016; Shu et al., 2018). In particular, IL-17 promotes the migration of neutrophils to the inflamed sites for the early clearance of bacteria by inducing the production of the chemokines CXCL1 and CXCL5 (Shen and Chen, 2018). In the present study, the expression of Th17-related mRNA showed that IL-23 and IL-6 were significantly activated early during infection, while IL-1β and IL-17 were highly activated after 6 h post infection. Comparison analysis showed that IL-6, IL-23, IL-17A, IL-17F, ROR*γ*T, and IL-22 were commonly activated in Th17-related signaling pathways.

In the Th1-diminished condition, IL-17 from Th17 cells is essential for inducing mature granuloma formation according to the Th17 cell immune response balance (Yoshida et al., 2010). In the present study, CSF2, CSF3, IL-22, IL-17A, IL-17F, CCL3, and CCL4 were significantly activated after 24 h.p.i. in the ‘Th17 Activation Pathway’ and ‘Differential Regulation of Cytokine Production in Macrophages and T Helper Cells by IL17A and IL17F’ pathways. Cytokine analysis also showed IL-17 and IL-1β were highly expressed compared to other cytokines at 24 h.p.i. IL-17 is also known to inhibit the apoptosis of *Mycobacterium*-infected macrophages to promote intracellular growth (Vázquez et al., 2012; Cruz et al., 2015a). In these studies, IL-17A was reported to inhibit p53 of the intrinsic apoptotic pathway by increasing BCL2 levels and decreasing BAX expression, CASP 3 activity, and cytochrome c release. Apoptosis is a bactericidal mechanism in infected host cells; however, *Mycobacterium* survives and replicates within macrophages by preventing apoptosis through several mechanisms (Rojas et al., 1998; Chen et al., 2006; O’Sullivan et al., 2007). In particular, *M. avium* was recently reported to inhibit bacterial programmed cell death induced by both the extrinsic pathway though caspase 8 and the intrinsic apoptotic pathway through caspase 3 (Sharbati et al., 2011; Kabara and Coussens, 2012).

In the analysis of signaling pathways, apoptosis signaling was inhibited at all times of infection. The BAX-CYCS-CASP9-CASP3/CASP7 signaling pathway was particularly inhibited at 24 h. The abundance of *caspase 3*, *caspase8*, *caspase9*, and *bax* after 24 h was also downregulated in the observed gene expression profiles. Furthermore, the activity of caspase 3/7 decreased over time in canine monocyte-derived macrophages infected with *M. avium*. In addition, *M. avium* were internalized into macrophages (26.5%), and the number of intracellular *M. avium* cells was significantly increased during infection over time. These results may indicate that *M. avium* replicates in canine macrophages by preventing apoptosis. However, caspase activity was not significantly down regulated and genes related to apoptosis signaling were significantly increased at the early time of infection, although they were decreased compared to that observed in uninfected cells after 24 h. Therefore, additional studies are needed to elucidate the mechanism of apoptosis inhibition after latent *M. avium* infection.

Although *M. avium* infection in dogs has increased, canine immune responses to *M. avium* have not been studied. In the present study, transcriptome analysis results showed that canine peripheral blood mononuclear cells expressed genes associated with the activation of the Th1 and Th17 responses and the inhibition of apoptosis in response to *M. avium* infection. In addition, intracellular *M. avium* cells were observed to replicate in canine monocyte-derived macrophages. These results could be related to the case reports of *M. avium*-infected dogs that showed granulomatous inflammation in infected tissues. These results might reveal why *M. avium* infection in dogs has continuously been reported although they are known to be resistant to members of the *Mycobacterium avium* complex. However, additional studies are needed to assess whether *M. avium* inhibits apoptosis and induces the proliferation of Th17 cells during long-term infections. Nevertheless, the results of our present study will help to identify the host responses against *M. avium* in various species and understand the immune response toward *M. avium* in infected dogs.

## Data Availability Statement

Raw files and normalized datasets are available from Gene Expression Omnibus (GEO) https://www.ncbi.nlm.nih.gov/geo under the accession number GSE133326.

## Ethics Statement

The Animal study was reviewed and approved by the Institutional Animal Care and Use Committee (IACUC) at GENIA (IACUC number; ORIENT-IACUC-19026). Blood samples were collected from six healthy Beagle dogs by professional veterinarians in accordance with the Guide for the Care and Use of Laboratory Animals and the Animal Welfare Act in the animal facility at Genia (Eumsung, Korea). The study with M. avium and the blood samples was reviewed and approved by the Seoul National University Institutional Biosafety Committee (protocol: SNUIBC-R180912-3).

## Author Contributions

Conceived and designed the experiments: SK, H-EP, and HY. Performed the experiments: SK, WP, S-YK, and H-TP. Analyzed the data: SK and HY. Correction and discussion: SK and HY. Wrote the paper: SK and HY. All authors contributed to the article and approved the submitted version.

## Funding

This study was carried out with the support of the “Cooperative Research Program of Center for Companion Animal Research (Project No. PJ01398501)” Rural Development Administration, Republic of Korea.

## Conflict of Interest

The authors declare that the research was conducted in the absence of any commercial or financial relationships that could be construed as a potential conflict of interest.
