# Supplementary material for: Mycobacterium avium Modulates the Protective Immune Response in Canine Peripheral Blood Mononuclear Cells
Source: Front Cell Infect Microbiol. 2021 Jan 14;10:609712. doi: 10.3389/fcimb.2020.609712 (PMC7840563; doi:10.3389/fcimb.2020.609712)
Supplement: Supplementary file 1 [file DataSheet_1.docx]

Supplementary Material

**Supplementary Table 1. Nucleotide Sequences of Primers.**

| **Gene** | **Forward sequence (5'→3')** | **Reverse sequence (5'→3')** | **Reference** |
| --- | --- | --- | --- |
| **IL-13** | TGATCAATGTCTCCGACTGC | ACAGTGCTTTCAGCATCCTCT | Pujol, Myriam, et al. (2017) |
| **IL-17** | GCTCCCCAGAGCAGACTTT | AAGAACCCTAATGAGTTTAGTCAGAAA |  |
| **IL-1β** | TACCTGTGGTCTTGGGCATC | TCTAGCTGTAGGGTGGGCTT |  |
| **IL-4** | GCTTACTAGCACTCACCAGCA | TCGTTTCTCGCTGTGAGGATG |  |
| **IL-23** | GACTCACAGAACGGACAGCA | TCAAATCTGGCTGGCTCTGG |  |
| **IFN-γ** | GTTGCTGCCTACTTGGGAAC | GGCGTCTGACATGCCTCTA |  |
| **IL-5** | ACCTGCAAGTATTTCTTGGTGTAA | AAGCCGGTTTGTTCTCAACTT |  |
| **IL-6** | TGGCTACTGCTTTCCCTACC | TTGAAGTGGCATCATCCTTG |  |
| **TNF-α** | TCACTTCCTCTGACCCCTCA | AGCCCTGAGCCCTTAATTCT |  |
| **TGF-β1** | TACATTGACTTCCGCAAGGA | GTTAGCGTGGTAACCCTTGG |  |
| **IL-10** | GCACCCTACTTGAGGACGAC | AGCTCTCGGAGCATGTGG |  |
| **IL-12p35** | CAGAGCAACAGATGGAGCAA | TTATTAACTCCATTCAAAAGCAACTG |  |
| **GAPDH** | GATGGGCGTGAACCATGAGA | TGGTCATGGATGACTTTGGCT |  |
| **CSF3** | TTCCTGGAGCTGGCATATCG | CAAGAGTGCAGGGCTCCTTT | XM_022423955.1 |
| **F3** | GCACCAGCCACGAGAAAGGTAT | GCTCCAAGGGCACCTTCTTTA | NM_001024640.1 |
| **HBEGF** | GACTCTCCCACCGAATCCAC | TGGCTTGGAGGAGAAAGCAG | XM_005617276.3 |
| **PTGS2** | GAGCACGCCTCGGGAACT | TCGCCGTAGAATCCTGTTCG | NM_001003354.1 |
| **SELENOP** | CCCAGCAATGTGGAGAAGCCT | GGCTTGAAGAAGAGCAACCAC | NM_001115118.1 |
| **SUCNR1** | TTTGTTGTGGGAGTCTTTGGGA | GCATGGGAAGGGTGCACAAA | XM_022408748.1 |
| **CXCL8** | GACTTCCAAGCTGGCTGTTG | GGGCCACTGTCAATCACTCT | NM_001003200.1 |
| **RORa** | GGCTTCTTTCCCTACTGTTCTT | CAGAATATATCTAAATCACATCTG | Kol, Amir, et al. (2016) |
| **RORc** | CTTACAATGCTGACAACCACAC | CATCTTTGACTTCTCCCGCT |  |
| **IL17A** | CAATGAGGACCCTGAGAGATAC | GACGGAGTTCATGTGGTAGTT |  |
| **IL17F** | AGTGTGAGGGTTGACATTCG | GTCGCGGGTAATGTTGTAGT |  |
| **CCR6** | TGTCCTCACTCTCCCATTCT | AGTTGAAGTTGATGGCGTAGAT |  |
| **IL23R** | CACAGACTACAAGGCGGAAA | TTGTGTATATTCCTGGTCTCAGC |  |
| **Condition** | **95°C for 3 min, followed by 40 cycles of 95°C for 3 s and 60°C for 30 s** | | |
| **OAZ1** | CACGGGCTCCAAACACATTA | TCATCGCGGTTCTTATGGAA | Herrmann, Ina, et al. (2018) |
| **IDO1** | GCACCGAGCCCATAAAGAGTT | GAGTTGCCTTTCCAACCAGAC |  |
| **CXCL11** | AGTGTGAAGGGCATGGTTACA | CCTTTGAACATGGGGAAATCTTG |  |
| **CD163** | GCGGCTTACAGTTTCCTTGAG | AGACACAGAAATTAGCCCAGCA |  |
| **CCL22** | ACTACATCCGTCACCCTCTG | TGACAGTTAGGAAGACCACGC |  |
| **LCN2** | AGCTGAAAGATGACCAGAGCTACAA | TACTCCAGGGTAAAGCTGAATGTCG |  |
| **CASP3** | TTCATTATTCAGGCCTGCCGAGG | TTCTGACAGGCCATGTCATCCTCA | Del Puerto, H. L., et al. (2010) |
| **CASP8** | ACAAGGGCATCATCTATGGCTCTGA | CCAGTGAAGTAAGAGGTCAGCTCAT |  |
| **CASP9** | TCAGTGACGTCTGTGTTCAGGAGA | TTGTTGATGATGAGGCAGTAGCCG |  |
| **BAX** | TTCCGAGTGGCAGCTGAGATGTTT | TGCTGGCAAAGTAGAAGAGGGCAA |  |
| **BCL2** | CATGCCAAGAGGGAAACACCAGAA | GTGCTTTGCATTCTTGGATGAGGG |  |
| **Condition** | **50°C for 2 min and 95°C for 10 min, followed by 40 cycles of 95°C for 15 s and 60°C for 60 s** | | |

**Supplementary Table 2. Top 20 Canonical Pathways in Canine Peripheral Blood Mononuclear Cells Infected with *Mycobacterium avium* for 6 h.**

| Ingenuity Canonical Pathways | -log  (*p*-value) | Ratio | z-score |
| --- | --- | --- | --- |
| Atherosclerosis Signaling | 12.4 | 0.408 | - |
| Hepatic Fibrosis / Hepatic Stellate Cell Activation | 10.9 | 0.337 | - |
| Agranulocyte Adhesion and Diapedesis | 10.5 | 0.332 | - |
| HMGB1 Signaling | 9.62 | 0.337 | 2.92 |
| Granulocyte Adhesion and Diapedesis | 9.43 | 0.326 | - |
| LXR/RXR Activation | 9.04 | 0.359 | -1.897 |
| Axonal Guidance Signaling | 8.69 | 0.243 | - |
| Hepatic Cholestasis | 8.54 | 0.307 | - |
| Cardiac Hypertrophy Signaling (Enhanced) | 7.24 | 0.232 | 3.763 |
| Role of Macrophages, Fibroblasts and Endothelial Cells in Rheumatoid Arthritis | 7.23 | 0.257 | - |
| Acute Phase Response Signaling | 7.2 | 0.298 | 1.852 |
| IL-10 Signaling | 7.03 | 0.397 | - |
| IL-6 Signaling | 6.75 | 0.325 | 3.781 |
| Role of Hypercytokinemia/hyperchemokinemia in the Pathogenesis of Influenza | 6.35 | 0.465 | - |
| STAT3 Pathway | 6.2 | 0.309 | 0.756 |
| LPS/IL-1 Mediated Inhibition of RXR Function | 6.11 | 0.266 | 0.853 |
| G-Protein Coupled Receptor Signaling | 5.49 | 0.246 | - |
| NF-κB Signaling | 5.33 | 0.272 | 3.063 |
| cAMP-mediated signaling | 5.31 | 0.255 | 2.023 |
| PPAR Signaling | 5.3 | 0.317 | -4.747 |

**Supplementary Table 3. Top 20 Canonical Pathways in Canine Peripheral Blood Mononuclear Cells Infected with *Mycobacterium avium* for 12 h.**

| Ingenuity Canonical Pathways | -log  (p-value) | Ratio | z-score |
| --- | --- | --- | --- |
| Atherosclerosis Signaling | 12.9 | 0.368 | - |
| Granulocyte Adhesion and Diapedesis | 9.3 | 0.282 | - |
| Role of Macrophages, Fibroblasts and Endothelial Cells in Rheumatoid Arthritis | 8.81 | 0.232 | - |
| Osteoarthritis Pathway | 8.78 | 0.259 | 2.449 |
| Hepatic Cholestasis | 8.21 | 0.261 | - |
| IL-6 Signaling | 7.98 | 0.302 | 3.124 |
| IL-10 Signaling | 7.94 | 0.37 | - |
| Axonal Guidance Signaling | 7.92 | 0.201 | - |
| HMGB1 Signaling | 7.72 | 0.271 | 3.413 |
| Cardiac Hypertrophy Signaling (Enhanced) | 7.65 | 0.198 | 2.251 |
| Acute Phase Response Signaling | 7.4 | 0.26 | 1.48 |
| Differential Regulation of Cytokine Production in Macrophages and T Helper Cells by IL-17A and IL-17F | 7.35 | 0.667 | - |
| Role of Pattern Recognition Receptors in Recognition of Bacteria and Viruses | 7.25 | 0.271 | 2.828 |
| Role of Hypercytokinemia/hyperchemokinemia in the Pathogenesis of Influenza | 7.23 | 0.442 | - |
| Role of Osteoblasts, Osteoclasts and Chondrocytes in Rheumatoid Arthritis | 7 | 0.238 | - |
| Altered T Cell and B Cell Signaling in Rheumatoid Arthritis | 6.94 | 0.322 | - |
| LXR/RXR Activation | 6.75 | 0.281 | -3.053 |
| Toll-like Receptor Signaling | 6.75 | 0.338 | 1.528 |
| Hepatic Fibrosis / Hepatic Stellate Cell Activation | 6.71 | 0.247 | - |
| Differential Regulation of Cytokine Production in Intestinal Epithelial Cells by IL-17A and IL-17F | 6.7 | 0.565 | - |

**Supplementary Table 4. Top 20 Canonical Pathways in Canine Peripheral Blood Mononuclear Cells Infected with *Mycobacterium avium* for 24 h.**

| Ingenuity Canonical Pathways | -log  (p-value) | Ratio | z-score |
| --- | --- | --- | --- |
| Atherosclerosis Signaling | 16.9 | 0.536 | - |
| Hepatic Fibrosis / Hepatic Stellate Cell Activation | 15.5 | 0.453 | - |
| LXR/RXR Activation | 14.9 | 0.508 | -3.742 |
| Axonal Guidance Signaling | 12.1 | 0.327 | - |
| Cardiac Hypertrophy Signaling (Enhanced) | 11.6 | 0.323 | -1.315 |
| Osteoarthritis Pathway | 10.3 | 0.382 | -0.471 |
| Role of Osteoblasts, Osteoclasts and Chondrocytes in Rheumatoid Arthritis | 8.73 | 0.361 | - |
| Agranulocyte Adhesion and Diapedesis | 8.43 | 0.373 | - |
| Role of Macrophages, Fibroblasts and Endothelial Cells in Rheumatoid Arthritis | 8.34 | 0.329 | - |
| Granulocyte Adhesion and Diapedesis | 8.15 | 0.376 | - |
| Differential Regulation of Cytokine Production in Macrophages and T Helper Cells by IL-17A and IL-17F | 8.07 | 0.833 | - |
| Colorectal Cancer Metastasis Signaling | 7.82 | 0.34 | -2.458 |
| LPS/IL-1 Mediated Inhibition of RXR Function | 7.78 | 0.349 | 0.756 |
| Differential Regulation of Cytokine Production in Intestinal Epithelial Cells by IL-17A and IL-17F | 7.68 | 0.739 | - |
| Regulation of the Epithelial-Mesenchymal Transition Pathway | 7.53 | 0.361 | - |
| Role of Cytokines in Mediating Communication between Immune Cells | 7.07 | 0.519 | - |
| FXR/RXR Activation | 6.56 | 0.38 | - |
| Ovarian Cancer Signaling | 6.4 | 0.373 | 0.209 |
| Role of Hypercytokinemia/hyperchemokinemia in the Pathogenesis of Influenza | 6.23 | 0.535 | - |
| Human Embryonic Stem Cell Pluripotency | 6.11 | 0.369 | - |

**Supplementary Table 5. Differentially Expressed Genes of ‘Th17 Activation Pathway’ in Canine Peripheral Blood Mononuclear Cells Infected with *Mycobacterium avium*.**

| Symbol | Entrez Gene Name | 6h | | 12h | | 24h | |
| --- | --- | --- | --- | --- | --- | --- | --- |
|  |  | FC | p-value | FC | p-value | FC | p-value |
| BATF | basic leucine zipper ATF-like transcription factor | 2.385 | 0.033 | - | - | -2.167 | 0.0874 |
| CCL20 | C-C motif chemokine ligand 20 | 2.41 | 0.0571 | - | - | - | - |
| CSF2 | colony stimulating factor 2 | - | - | 14.046 | 0.312 | 235.871 | 0.355 |
| DEFB110 | defensin beta 110 | - | - | 2.338 | 0.416 | - | - |
| DEFB114 | defensin beta 114 | 2.44 | 1 | - | - | -2.013 | 0.536 |
| DEFB118 | defensin beta 118 | 2.274 | 0.19 | - | - | - | - |
| DEFB124 | defensin beta 124 | - | - | 3.4 | 0.424 | 3.021 | 0.0761 |
| DEFB125 | defensin beta 125 | 2.469 | 0.00123 | - | - | 3.226 | 0.321 |
| DEFB128 | defensin beta 128 | - | - | 2.497 | 0.111 | - | - |
| IFNG | interferon gamma | 4.854 | 0.238 | 3.133 | 0.193 | 2.638 | 0.583 |
| IL6 | interleukin 6 | 151.151 | 0.0123 | 76.046 | 0.0879 | 63.558 | 0.26 |
| IL10 | interleukin 10 | - | - | - | - | 5.965 | 0.25 |
| IL21 | interleukin 21 | - | - | - | - | 3.483 | 0.106 |
| IL22 | interleukin 22 | - | - | 5.035 | 0.151 | 125.256 | 0.196 |
| IL12A | interleukin 12A | - | - | - | - | -7.231 | 0.346 |
| IL12B | interleukin 12B | 3.398 | 0.0144 | 3.034 | 0.00755 | 5.301 | 0.148 |
| IL12RB1 | interleukin 12 receptor subunit beta 1 | 2.117 | 0.0139 | - | - | - | - |
| IL17A | interleukin 17A | 64.059 | 0.00997 | 31.612 | 0.065 | 164.794 | 0.196 |
| IL17F | interleukin 17F | 3.306 | 0.127 | 18.132 | 0.279 | 50.502 | 0.0022 |
| IL1B | interleukin 1 beta | 10.655 | 0.211 | 39.655 | 0.0194 | 30.245 | 0.0152 |
| IL1R1 | interleukin 1 receptor type 1 | 2.498 | 0.159 | - | - | - | - |
| IL21R | interleukin 21 receptor | 2.478 | 0.0498 | - | - | - | - |
| IL23A | interleukin 23 subunit alpha | - | - | - | - | 4.233 | 0.177 |
| IL23R | interleukin 23 receptor | - | - | -2.148 | 0.227 | -3.806 | 0.518 |
| IRAK2 | interleukin 1 receptor associated kinase 2 | 6.554 | 0.0517 | 3.262 | 0.102 | 2.55 | 0.222 |
| IRAK3 | interleukin 1 receptor associated kinase 3 | - | - | 2.799 | 0.00694 | 3.45 | 0.0178 |
| IRF4 | interferon regulatory factor 4 | - | - | - | - | 2.064 | 0.00668 |
| NFATC4 | nuclear factor of activated T cells 4 | -3.305 | 0.424 | 11.138 | 0.127 | -8.387 | 0.22 |
| NFKB1 | nuclear factor kappa B subunit 1 | 2.324 | 0.106 | - | - | 2.237 | 0.0477 |
| NFKB2 | nuclear factor kappa B subunit 2 | 3.807 | 0.00829 | 4.007 | 0.00819 | 2.637 | 0.027 |
| PTGER2 | prostaglandin E receptor 2 | 5.519 | 0.0145 | - | - | 2.841 | 0.141 |
| PTGER4 | prostaglandin E receptor 4 | - | - | - | - | -2.443 | 0.167 |
| RELA | RELA proto-oncogene, NF-kB subunit | - | - | 2.062 | 0.0146 | - | - |
| RORA | RAR related orphan receptor A | - | - | - | - | -2.115 | 0.555 |
| RORC | RAR related orphan receptor C | 6.997 | 0.398 | 2.582 | 0.577 | 4.191 | 0.354 |
| SOCS3 | suppressor of cytokine signaling 3 | 3.562 | 1 | 5.803 | 0.422 | - | - |

**Supplementary Table 6. Differentially Expressed Genes of ‘Differential Regulation of Cytokine Production in Macrophages and T Helper Cells by IL17A and IL17F’ in Canine Peripheral Blood Mononuclear Cells Infected with *Mycobacterium avium*.**

| Symbol | Entrez Gene Name | 6h | | 12h | | 24h | |
| --- | --- | --- | --- | --- | --- | --- | --- |
|  |  | FC | p-value | FC | p-value | FC | p-value |
| CCL2 | C-C motif chemokine ligand 2 | - | - | - | - | 6.555 | 0.0171 |
| CCL3 | C-C motif chemokine ligand 3 | 24.603 | 0.186 | 14.776 | 0.166 | 76.672 | 0.0894 |
| CCL4 | C-C motif chemokine ligand 4 | 17.036 | 0.0929 | 12.157 | 0.164 | 39.417 | 0.0952 |
| CCL5 | C-C motif chemokine ligand 5 | - | - | 2.095 | 0.0512 | 2.657 | 0.0425 |
| CSF2 | colony stimulating factor 2 | - | - | 14.046 | 0.312 | 235.871 | 0.355 |
| CSF3 | colony stimulating factor 3 | 296.498 | 0.00161 | 42.335 | 0.0175 | 2712.837 | 0.352 |
| IL6 | interleukin 6 | 151.151 | 0.0123 | 76.046 | 0.0879 | 63.558 | 0.26 |
| IL9 | interleukin 9 | -4.908 | 0.423 | - | - | - | - |
| IL10 | interleukin 10 | - | - | - | - | 5.965 | 0.25 |
| IL13 | interleukin 13 | 3.523 | 1 | 10.015 | 0.171 | 3.486 | 0.471 |
| IL12A | interleukin 12A | - | - | - | - | -7.231 | 0.346 |
| IL12B | interleukin 12B | 3.398 | 0.0144 | 3.034 | 0.00755 | 5.301 | 0.148 |
| IL17A | interleukin 17A | 64.059 | 0.00997 | 31.612 | 0.065 | 164.794 | 0.196 |
| IL17F | interleukin 17F | 3.306 | 0.127 | 18.132 | 0.279 | 50.502 | 0.0022 |
| IL1B | interleukin 1 beta | 10.655 | 0.211 | 39.655 | 0.0194 | 30.245 | 0.0152 |
| TNF | tumor necrosis factor | 13.068 | 0.0771 | 7.568 | 0.00275 | 8.211 | 0.107 |

**
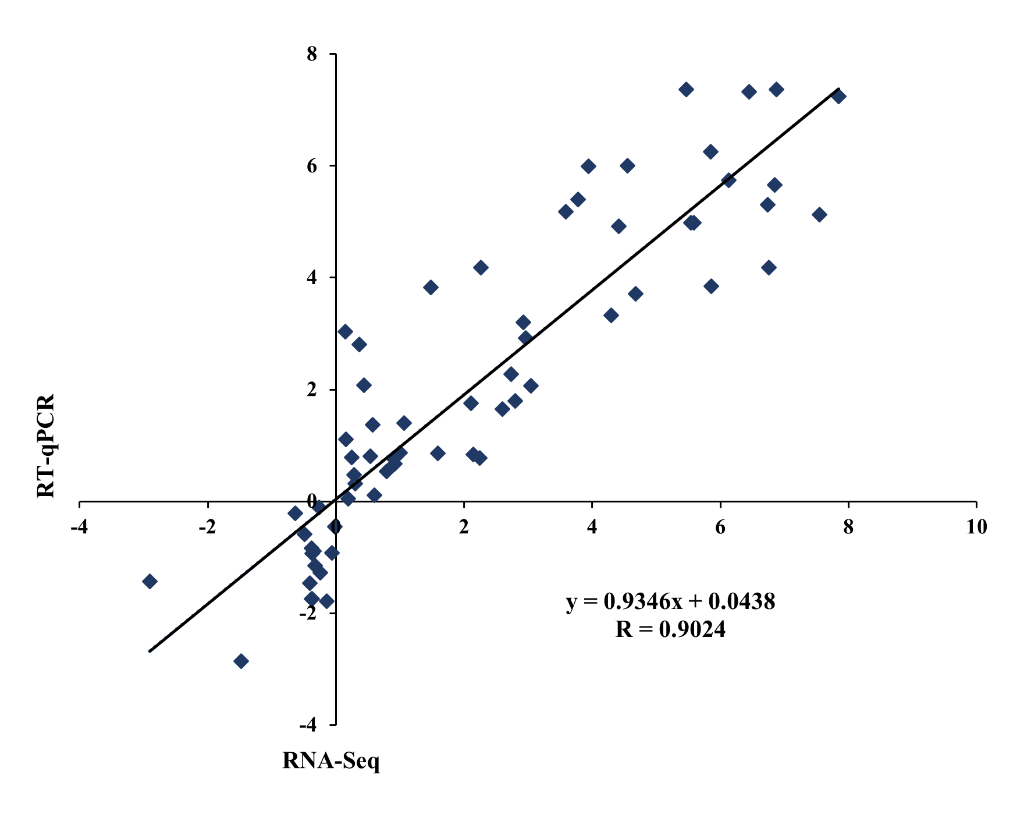
**

**Supplementary Figure 1. Validation of Gene Expression by RNA-Seq and Quantitative Real-Time PCR.** The relative expression level was compared to that observed in the control cells to determine the fold change in expression for each gene.


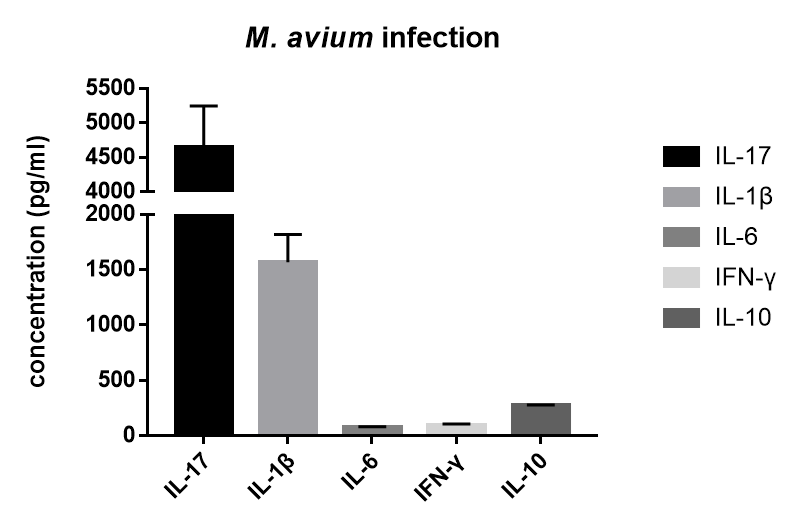


**Supplementary Figure 2. Cytokine Expression in Canine Peripheral Blood Mononuclear Cells Infected with *Mycobacterium avium* at 24 hpi.** Supernatants were analyzed for IL-17, IL-1β, IL-6, IFN-γ, IL-10, IL-4, and IL-12 by ELISA. IL-4 and IL-12 were not detected. Each bar represents the mean ± SD of independent experiments (n = 6).


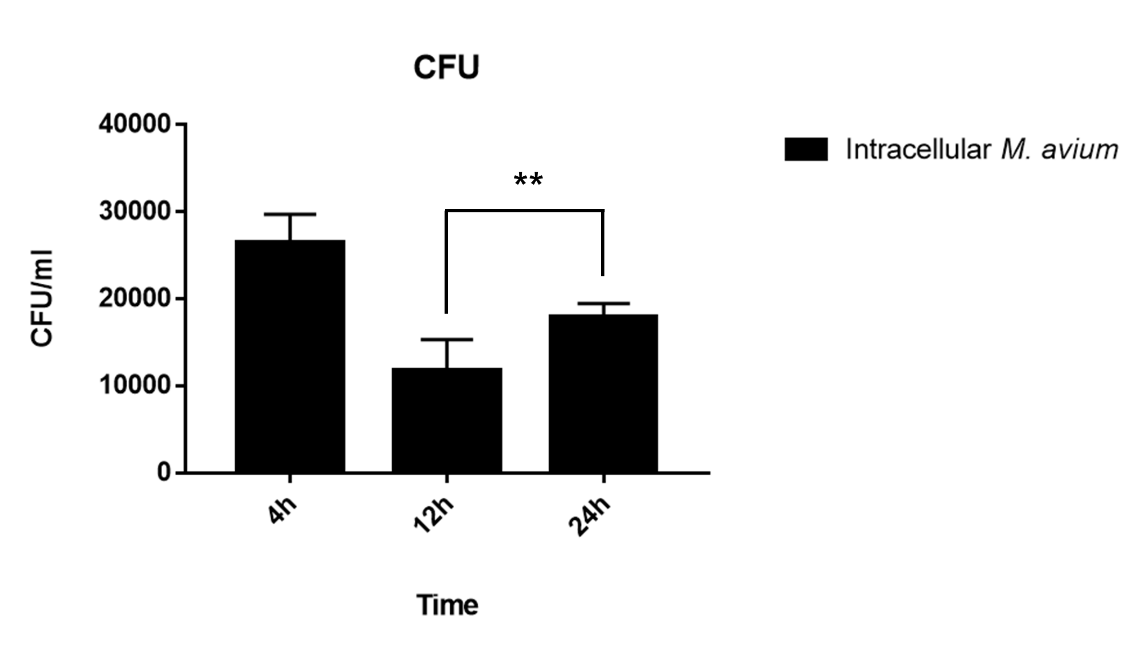


**Supplementary Figure 3. The number of intracellular *Mycobacterium avium* in canine monocyte-derived macrophages.** Graph showing the number of intracellular *M. avium* cells in MDMs after treatment with amikacin. Each column represents the mean ± SD of nine independent experiments. **$p<0.01$.
